# Novel and Conservative Approaches Towards Effective Management of Plantar Fasciitis

**DOI:** 10.7759/cureus.913

**Published:** 2016-12-05

**Authors:** Salman Assad, Awaiz Ahmad, Immad Kiani, Usman Ghani, Vikram Wadhera, Todd N Tom

**Affiliations:** 1 Department of Medicine, Shifa Tameer-e-Millat University, Islamabad, Pakistan; 2 Neurosurgery, Shifa International Hospital, Islamabad, Pakistan; 3 Internal Medicine, Shifa International Hospital, Islamabad, Pakistan; 4 Department of Medicine, Shifa International Hospital, Islamabad, Pakistan; 5 Department of Surgery, Mount Sinai Health System; 6 Surgery, ARH Regional Healthcare Center/University of Kentucky College of Medicine

**Keywords:** plantar fasciitis, physiotherapy, treatment, corticosteroids, botulinum type a toxin

## Abstract

We assessed the effectiveness of the different treatments for plantar fasciitis (PF) based on the changes in functional outcomes. A systematic literature search was carried out and studies from 2010 to 2016 were included in this review. The databases from Google Scholar, PubMed and Cochrane were used for the various treatment modalities of plantar fasciitis. The objectives measured included visual analog scale (VAS), Roles and Maudsley scale, foot function index (FFI), plantar fascia thickness and American Orthopedic Foot and Ankle Society (AOFAS) ankle-hind foot scale as the tools to predict the improvement in symptoms of pain and discomfort. Eight randomized controlled trails that met the selection criteria were included in this review. Extracorporeal shock wave lithotripsy (ESWL) with botulinum toxin type A, corticosteroid injections, autologous whole blood and plasma treatment, novel treatments like cryopreserved human amniotic membrane, effect of placebo, platelet rich plasma injections and corticosteroid injections, physiotherapy and high strength training were analyzed. All the treatment modalities applied did lead to the reduction in pain scores, but for long term management autologous condition plasma and platelet rich plasma are the preferred treatment options. Impact of physiotherapy and high strength training is equivalent to corticosteroid injections and hence is suited for patients avoiding invasive forms of treatment.

## Introduction and background

Plantar fasciitis (PF) is a problem that is affecting a majority of people who are either involved in sports or work long periods standing. Just in the United States alone, it affected one million people per year between the years 1995-2000 [1]. In simple terms, plantar fasciitis involves pain and inflammation in a thick band of tissue in the body. This band known as the plantar fascia runs from the bottom of the foot and connects our toes with the heel bone. Some of the common risk factors of this include prolonged standing, obesity, excessive foot pronation, running, and decreased ankle dorsiflexion [2].

The symptoms of plantar fasciitis are like stabbing pain with the very first steps in the morning. Once the foot is on the go and walking, the pain usually gets better. However, this pain is likely to return from long periods of standing or getting up from a seated position. The etiology of PF is multifactorial and that explains the multiple treatment modalities [3]. The cause basically includes inflammation and degeneration of the plantar fascia origin. The fascia normally works like a shock-absorbing bowstring. In other words, it supports the arch of the foot. There are many treatment modalities for plantar fasciitis: non-invasive (physiotherapy) to invasive (surgery and corticosteroid injections). These days, the treatment options available include autologous plasma transfusions, corticosteroid injections, physiotherapy-like strength training, and extracorporeal shock wave therapy (ESWT) [3].

Conservative treatments have always been the first approach for treating PF and are actually used in 85 to 90% of the cases [4]. The main conservative treatments recommended include nonsteroidal anti-inflammatory drugs (NSAIDs), prefabricated orthotics and stretching for at least six to twelve months. Many have recommended that shock wave therapy has long-term results than steroid injections. Extracorporeal shock wave therapy (ESWT) reduced pain and improved function in 34% to 88% of the cases. ESWT is comparable to surgical plantar fasciotomy without any operative risks and yields good long-term effects [5]. We have done this review to analyze the various efficacies of various treatment options offered for plantar fasciitis. This review assesses the effectiveness of the different treatments for plantar fasciitis (PF) based on the changes in outcomes such as plantar fascia thickness, VAS score and other foot pain related scales and questionnaires.

## Review

### Materials & methods

**Figure 1:**
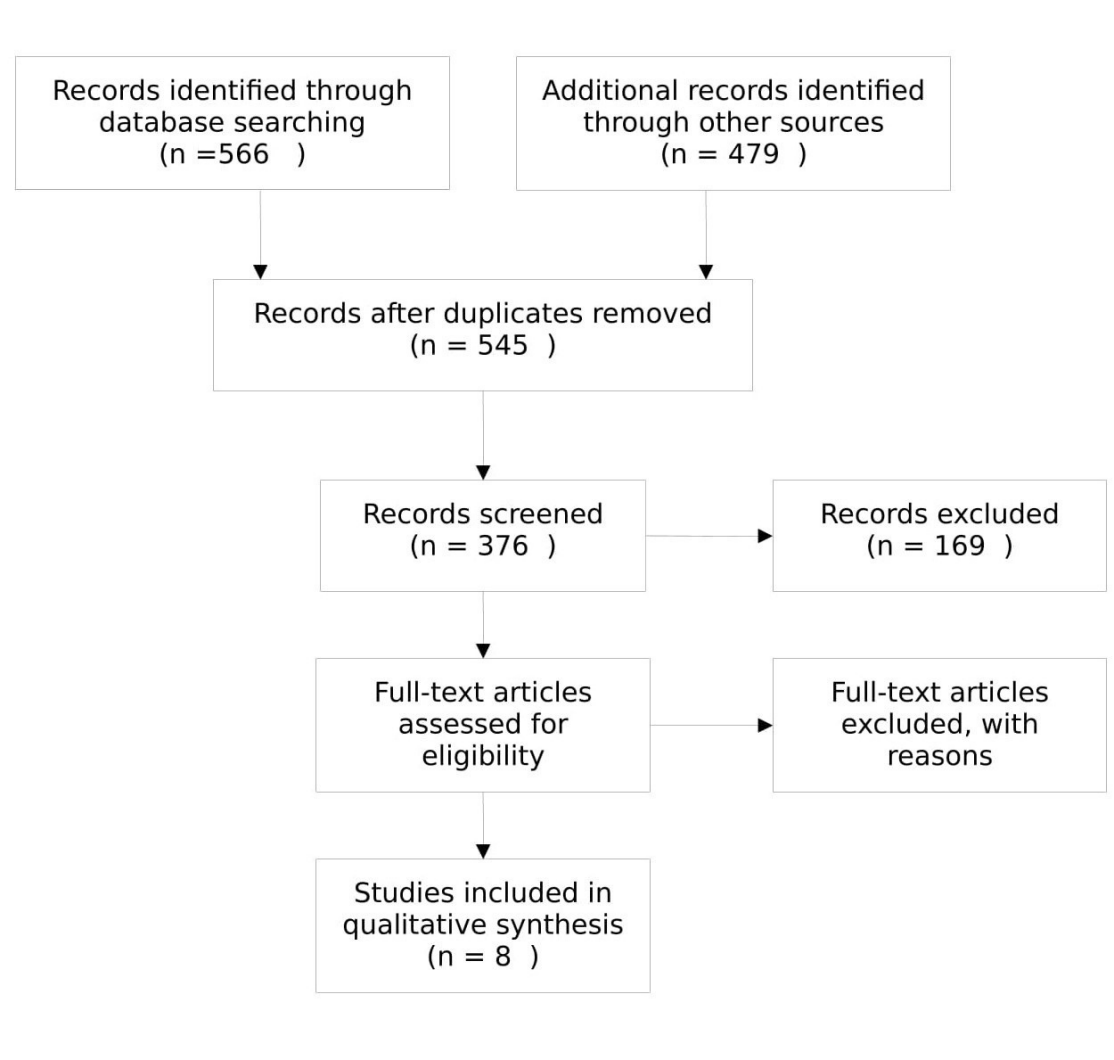
Flow chart of selection of studies

The literature was searched for the studies that reviewed the effect of various treatments on the improvement in symptoms of plantar fasciitis. The objectives measured included VAS and AOFAS as the tools to predict the improvement in symptoms of pain and discomfort. We have carried out an extensive review of the literature on Google scholar, PubMed and Cochrane review for the various treatment modalities of plantar fasciitis. The studies that were used are dated after 2010 and thus are in the last five years (Figure1). The keywords that were utilized in this search consisted of “Plantar Fasciitis” “Treatment” “Physiotherapy” and “most effective.” The studies that were determining the efficacy and the cost-effectiveness of the treatment were included in this review.

Inclusion Criteria

The inclusion criteria were the comparison of one or two modalities of treatment for plantar fasciitis. The respondents in the study must not have other systemic diseases and must have the problem for more than six months.

Exclusion Criteria

The exclusion criteria for the studies were that the patients must have chronic plantar fasciitis or persistent pain despite the use of conventional treatments.

Objectives Assessed

**Figure 2:**
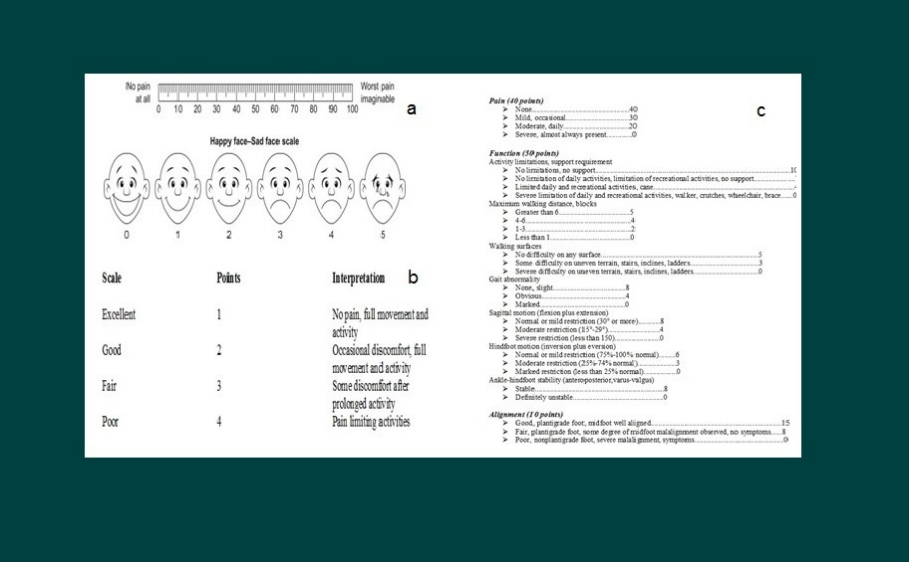
Tools to predict the improvement in symptoms (VAS, RM Scale, AOFAS) Visual Analogue Scale (VAS) _(2a)_ Roles and Maudsley (RM) Scale _(2b)_ American Orthopaedic Foot and Ankle Society (AOFAS) Scale _(2c)_

1. Visual analog scale & Roles and Maudsley scale: The orthopedic pain scale includes a simple 10 point visual analog scale (VAS) which ranges from a scale of either one to 10 or one to 100 (Figure 2a) [6]. This score is often used in many studies in aiding clinician’s interpretation of pain. Basically, this is a measure that allows the patient to subjectively describe his or her pain and whether it has been augmented or relieved. Roles and Maudsley scale scores range from 0-4 points for excellent to poor (Figure 2b) [7].

2. American Orthopaedic Foot and Ankle Society (AOFAS) scale: The total score is set at 100 points. Each item included was based on both subjective and objective assessment and is scored from clinical observation and finding. Each of the four measures (i.e. those for the ankle-hindfoot, midfoot, hallux and lesser toes) were distributed into three major categories of pain, function and alignment. In addition, interpretation and criteria for scoring accompanied each item (Figure 2c) [8].

3. Foot function index (FFI): It is a measure of foot pain and disability. A foot function index (FFI) was made to analyze the effect of foot problems and pathologies on functional status in terms of pain, disability and restricted movements. The index consists of 23 items divided into three subscales or categories [9].

4. Fascia thickness: Heel pain is a common complaint and fascia thickness is one of the components used in the diagnosis of plantar fasciitis (Abul, et.al., 2015) [6]. It is noted that mean thickness of the plantar fascia measured by ultrasound is 0.3 mm in symptomatic heels as compared with 0.2 mm in asymptomatic heels. Ultrasound was used to measure plantar fascia thickness of 156 healthy adults with no previous complaints of heel pain. The overall mean thickness was 3 mm for both right and left feet. Nonetheless, plantar fascia thickness was less than 4 mm in the participants who did not have any clinically significant heel pathology. A study carried out by Cardinal, et al. (1996) revealed that the plantar fascia of the symptomatic feet was much thicker (mean 5.2 mm) than the fascia of asymptomatic feet (mean 2.9 mm) [10].

### Discussion

1. *Autologous Conditioned Plasma (ACP), Extracorporeal Shockwave Therapy (ESWT) & Conventional Treatment*

The Chew K, et al. study was carried out initially on 100 subjects but only 54 subjects agreed to participate [11]. Nineteen of the patients were taken into the autologous conditioned plasma (ACP) group whereas 19 were taken to the extracorporeal shockwave therapy (ESWT) group and 16 to the group of conventional treatment. Stretching exercises and orthotics, if needed, were included in the conventional treatment group. All three groups were comparable in age, gender, pain, and duration at the initiation of the study. There was a significant reduction in VAS pain score in all treatment groups from the base line to follow-up. The ESWT group revealed significant improvement in VAS pain scores at all the assessment time points as opposed to the conventional treatment group.

As for the American Orthopaedic Foot and Ankle Society (AOFAS) ankle hind-foot scale, the ACP group revealed a median improvement of 36 points, 28 points in the ESWT group and 15.5 points in the conventional treatment groups. The conventional treatment group had the lowest median change in all the different assessment points. As for plantar fascia thickness, all groups revealed a significant decrease in plantar fascia thickness at one and three months compared with baseline. Improvement in the ultrasound plantar fascia thickness at six months follow-up was 1.3 mm of the ACP group compared with improvements of 0.6 mm for both the ESWT and conventional groups. In comparison, ESWT and ACP are both considered better than conventional treatments when looking at the VAS scores, AOFAS ankle-hind foot scale scores and the decrease in ultrasound plantar fascia thickness. The objective improvement was more precedent in the ACP treatment group with the maximum decrease in ultrasound plantar fascia thickness. Alteration in plantar fascia thickness is a good objective measurement of the effectiveness of plantar fascia treatment. Overall, ACP injections have been considered the treatment of choice for objective and subjective improvement in the treatment of plantar fasciitis (Table 1).

**Table 1:** Conditioned plasma with conventional and extracorporeal shockwave therapy Plantar fascial thickness (PFT), American Orthopaedic Foot and Ankle Society (AOFAS) ankle hind foot scale, extracorporeal shockwave therapy (ESWT), autologous conditioned plasma (ACP), conventional treatment (CT)

	Visual analog scale			Decrease in PFT^*^ (P-value)			Improved AOFAS ankle hind foot scale^**^ (P value)		
	1 month	3 month	6 month	1 month	3 month	6 month	1 month	3 month	6 month
ACP ^††^ compared with CT^‡^	.037	-	-	.015	.014	-	-	.004	.013
ESWT^†^ compared with CT	.017	.022	.042	-	.019	.027	.011	.003	-

2. *Placebo, Corticosteroid Injection and Platelet Rich Plasma Proteins*

Mahindra P, et al. reviewed the comparison of placebo (normal saline given), corticosteroid injection and platelet-rich plasma injections [12]. The objectives reviewed in this study also consisted of VAS and AOFAS ankle scores. The mean VAS score decreased from 7.44 to 2.52 in the platelet-rich plasma group and from 7.72 to 3.64 in the corticosteroid group. As for the AOFAS score, there was a mean improvement in the score in the platelet-rich plasma group from 51.56 to 88.24 and the improvement in the corticosteroid group was from 55.72 to 81.32. No improvement was seen in the placebo group. However, there was a significant improvement in VAS scores and AOFAS scores at both three months and three weeks for both the groups. In comparison of the two modalities of treatment, the platelet-rich plasma injection was more effective than the corticosteroid group (Table 2).

**Table 2:** Platelet-rich plasma proteins, corticosteroid injection and placebo *American Orthopaedic Foot and Ankle Society (AOFAS), ** Platelet rich plasma proteins

Duration	Visual analogue scale pain scores			AOFAS^* ^score		
	PRP** group	Corticosteroid Injection group	Placebo group	PRP** group	Corticosteroid injection group	Placebo group
Pre-intervention	7.44	7.72	7.56	51.56	55.72	50.28
Three week – three months Follow-up	2.52	3.64	7.44	88.24	81.32	50.84

Tsikopoulos K, et al. compared the efficacy of corticosteroid injections vs. autologous whole blood in plantar fasciopathy [13]. The primary outcome being studied was pain relief and secondary outcome was the evaluation of composite results. The outcomes were assured after 2-6 weeks, 8-13 weeks and 24-16 weeks. The results revealed that for short term there was statistically significant difference in favor of corticosteroids; however, in medium term assessment the statistically significant difference was in favor of the autologous whole blood treatment. Therefore, this review deducted that corticosteroid is superior in relieving pain at two to six weeks, whereas autologous whole blood provides significant relief at 8-24 weeks.

In this study carried out by Aksahin E, et al., the effects of local injection of platelet rich plasma and corticosteroids in the treatment of chronic plantar fasciitis were compared [14]. The study included 60 patients who had plantar fasciitis for more than three months and did not have any response to the conventional treatment. The treatment groups were made such that the first 30 persons were included in the corticosteroid group and the other 30 were included in the platelet rich plasma (PRP) group. The first group was treated by local injection of 2 ml of 40 mg methylprednisolone with 2 ml of two percent prilocaine and the second group was treated with 3 ml of platelet rich plasma (PRP) after 2 ml of two percent of prilocaine injection. The objectives measured in this study were according to the modified criteria of Roles-Maudsley scores and visual analog scale pre-intervention at three months and at six months (Table 3).

**Table 3:** Corticosteroid injections and platelet-rich plasma treatment *Visual Analogue Scale (VAS), **Platelet Rich Plasma (PRP)

Duration	VAS^*^ pain scores	
	PRP^** ^group	Corticosteroid injection
Pre-intervention	7.33	6.2
Three weeks	--	---
Six months	3.93	3.4

After a review of these results, the study concluded that there was no significant difference between the PRP group and the steroid group at both three weeks and six months (P > 0.05). However, it was stated that the corticosteroid treatment does come with many other complications. For this reason, PRP injections were considered much safer treatment for chronic plantar fasciitis.

3. *ESWT and Botulinum Toxin Type A*

In another study by Roca B, et al., ESWT was compared with botulinum toxin type A in the treatment of plantar fasciitis [15]. This was a prospective randomized study in which 72 patients were included. The objectives viewed in this study also consisted of VAS for pain. In the group that received the ESWT, there was more improvement as opposed to those who received the botulinum toxin type A (BoNT-A). In the group receiving the ESWT, the median of improvement in VAS was two whereas in those receiving BoNT-A, the improvement was one. In a multivariate analysis, use of ESWT and lower weight was linked with improvement of pain in at least one of the pain scales. This study concluded that ESWT was both superior in control of pain and in long-term management of plantar fasciitis (Table 4).

**Table 4:** ESWT and botulinum toxin type A in terms of VAS and Roles and Maudsley scale Extracorporeal shock wave therapy (ESWT), botulinum toxin type A (BoNT-A), visual analogue scale (VAS), Roles and Maudsley (RM) Scale

Treatment	ESWT^*^	BoNT-A^**^	P- value
VAS ^†^ (median and interquartile range)	2 (1-4) points	1 (0-2) points	0.009
RM scale ^† †^ (median and interquartile range)	1 (0-1) points	0 (0-1) points	0.006

4. *Cryopreserved Human Amniotic Membrane Injection for Plantar Fasciitis: A Randomized, controlled, Double-blind Pilot Study*

This study discusses the use of a new treatment for chronic plantar fasciitis. A randomized controlled trial was initiated with two patient groups [16]. One patient group received an injection of the new cryopreserved human amniotic membrane (c-hAM) and the other group received an injection of corticosteroids. Corticosteroids have been previously used in the treatment of this condition; however, c-haM was tested the first time. Patients were randomized and received an injection at a baseline visit and then another injection at a six week follow-up. Total follow-up was obtained 12 weeks after the most recent injection. Outcomes were measured using the foot health status questionnaire (FHSQ) and the VAS scale. The results were dependent on whether the respondents came back for their second injections. Overall, there was no significant statistical difference between the two treatments. It should be noted that cryopreserved c-hAM had greater foot pain improvement in the two injection cohort. Considering that this was a pilot study, further investigation is required for the use of c-haM in the treatment of plantar fasciitis. Nonetheless, this treatment has been comparable to steroid injections and can be considered as an upcoming treatment for plantar fasciitis.

5. *Comparison of a Physiotherapy Program Versus Dexamethasone Injections for Plantar Fasciopathy in prolonged Standing Workers: A Randomized Clinical Trial*

Ryan M, et al. compared the use of dexamethasone injections and physiotherapy for chronic plantar fasciopathy in prolonged standing workers [17]. This study is of particular interest because of the comparison of invasive technique with noninvasive procedure. Patients were randomly divided into a PHYSIO group (exercises performed daily over a 12 week period and an INJECTION group (one palpation-guided dexamethasone injection followed by a daily calf stretching). Fifty six workers with greater than five hours/day standing with chronic plantar fasciopathy were included in this study. Both groups were seen for improvements in foot & ankle disability index (FADI) and visual analog scores for pain at work and with daily activities. There was improvement in baseline scores for both the groups but there was no significant change to plantar fascia thickness reported at six and twelve week follow-up points. Therefore, this study concluded that an injection of corticosteroid with stretching has the same therapeutic effectiveness as a physiotherapy-led exercise program.

6. *Shoe Inserts, High Load Strength Training and Plantar-Specific Stretching Exercise*

Rathleff MS, et al. compared the efficacy of shoe inserts and daily plantar-specific stretching as opposed to shoe inserts and high load progressive strength training in PF patients [18]. Forty eight patients with ultrasonographic diagnosed plantar fasciitis were randomized into stretch and strength groups. The stretch group (n=24) included routinely plantar-specific stretching exercise and shoe inserts while the strength group (n=24) consisted of shoe inserts and high load progressive strength exercise. The primary outcome was the foot function index (FFI) at three months, and follow-ups were performed at first, sixth and 12 months. The secondary outcomes were thickness of the plantar fascia, sports participation, and satisfaction with the results of treatment. At the primary endpoint of three months, FFI of the strength group was 29 points lower than 43 points of the stretch group [95% confidence interval (CI): 6–52, P = 0.016]. No disparity was found in secondary outcomes in between stretch and strength groups. However, no difference was noted at first, sixth and 12 months in primary outcome (Table 5).

**Table 5:** Shoe inserts and high load strength training Foot function index (FFI), confidence interval (CI)

Duration	FFI^a^ stretch group (n=24)	FFI strength group (n=24)	CI ^**^, P- value
Baseline	73	84	-
At three months	43	29	6–52, 0.016

The conclusion of this study was that simple progressive exercise protocol carried out every second day resulted in better self-reported outcome as opposed to plantar-specific training. Therefore, it has been recommended that high load strength training may lead to quicker reduction in pain and improvements in function for patients affected with chronic plantar fasciitis. A simple progressive exclusive protocol that consists of high load training performed every second day would result in quicker reduction and improvement.

### Limitations

The probability of selection bias in this review cannot be ignored. Language bias cannot be ignored because this review only included the literature published in the English language. Although every possible effort has been made to identify all relevant studies, some studies may have been missed due to limited access to research papers from databases and journals. There is also scarce evidence on cost-effectiveness of various treatment modalities for plantar fasciitis. Finally, this review is based on a modest body of evidence (eight randomized controlled trial (RCTs)), which were encountered by several methodological errors like small sample sizes.

## Conclusions

Review of these trials revealed that all the treatment modalities applied did lead to reduction in pain scores at some point. However, in comparison, long term relief was attained by autologous conditioned plasma and platelet rich plasma. Corticosteroid injections did provide pain relief but this treatment comes with a lot of side effects. Both corticosteroid injections and physiotherapy revealed that people avoiding invasive treatment can rely on physiotherapy for symptoms resolution. 
